# Risk of nonpulmonary infections requiring hospitalization in spondyloarthritis

**DOI:** 10.1002/iid3.615

**Published:** 2022-04-19

**Authors:** Ho Yin Chung, Shirley Chiu Wai Chan, Frances Sze Kei Sun

**Affiliations:** ^1^ Division of Rheumatology and Clinical Immunology The University of Hong Kong Hong Kong; ^2^ Chiron Medical Hong Kong

**Keywords:** infections, infliximab, psoriasis, spondyloarthritis, steroids, urinary tract infection

## Abstract

**Objectives:**

To compare the risk of five nonpulmonary infections leading to hospitalization between spondyloarthritis (SpA) and nonspecific back pain (NSBP), and to identify the risk factors.

**Methods:**

A total of 3018 patients with SpA and 2527 patients with NSBP were identified. Data from December 1995 to June 2019 was retrieved from a centralized electronic medical record system. The date of onset of five types of nonpulmonary infections including: urinary tract infection (UTI), skin infection, gastroenteritis (GE), septic arthritis, and pancreato‐hepatobiliary tract infection were identified. Demographic data, comorbidities, and medications used were also retrieved. Comparative risk of each type of infection between SpA and NSBP was determined using propensity score adjustment method. Cox regression model was used to identified risk factors.

**Results:**

Patients with SpA were younger in age, predominantly male, with fewer comorbid diabetes mellitus (DM), renal impairment, and depression. Compared with NSBP, patients with SpA had higher risk of UTI (hazard ratio [HR] 1.91; *p* < .001), skin infection (HR 1.79; *p* < .001), and septic arthritis (HR 4.57; *p* = .04). Risk of GE (HR 1.42; p = 1.00), and pancreato‐hepatobiliary tract infection (HR 1.67; *p* = .06) were not increased. Infliximab was an independent risk factor for UTI (HR 2.21; *p* = .04). Duration of steroid therapy >6 months (HR 2.22; *p* < .001), smoker (HR 1.81; *p* < .001), and psoriasis (HR 2.47; *p* < .001) were risk factors for skin infection.

**Conclusion:**

SpA was associated with increased risk of UTI, skin infection, and septic arthritis. Infliximab, prolonged steroid therapy, smoking, and psoriasis were associated risk factors.

## Graphical abstract

Spondyloarthritis was associated with an increased risk of urinary tract infection, skin infection, and septic arthritis that required hospitalization. The associated risk factors included Infliximab, prolong steroid therapy, smoking, and psoriasis. Rheumatologists should avoid prolong steroid therapy, and advise patients to quit smoking to prevent infections.

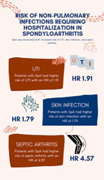

## 1 INTRODUCTION

Spondyloarthritis (SpA) is a spectrum of rheumatic diseases characterized by inflammation of the spine and sacroiliac (SI) joints, peripheral arthritis, and enthesitis. The prevalence of SpA varies across the world, ranging from 0.2% in southeast Asia to 1.6% in the Northern Arctic region.^1^ Ankylosing spondylitis (AS) is the prototypic disease, which predominantly affects young men. In contrast, there is no gender disparity in the prevalence of nonradiographic axial SpA. The treatment of SpA often includes the use of nonsteroidal anti‐inflammatory drugs (NSAIDs), conventional disease‐modifying antirheumatic drugs, and biologics (such as antitumor necrosis factor [anti‐TNF] and anti‐interleukin‐17 [anti‐IL17]).

SpA is associated with increased risk of comorbidities including cardiovascular events,^2^ metabolic syndrome,^3^ malignancies,^4^ and infections,^5^ all of which contribute to excess morbidity and mortality. Acquired immunodeficiency secondary to pharmacologic therapy may further increase the risk.^6^ Compared with data in rheumatoid arthritis (RA),^7^ this association is less well established in SpA. A meta‐analysis of randomized controlled trials (RCT)^8^ found no increased risk of infection in patients with ankylosing spondylitis (AS) on anti‐TNF therapy. However, generalizability was limited by the inclusion of relatively few studies in highly selective populations with short study durations. On the other hand, a series of recent observational studies found that disease‐modifying antirheumatic drugs (DMARDs) and prolonged use of steroids were associated with herpes zoster,^9^ and tuberculosis infection.^10^ A recent multicenter cohort demonstrated an increased risk of community‐acquired pneumonia in SpA compared with nonspecific back pain (NSBP).^11^ Further investigation using real‐life data to delineate the risk of other nonpulmonary infections is therefore important for the prevention of infection and guiding public policy.

We performed a retrospective study using a territory‐wide electronic healthcare database to identify the risk of the five most prevalent nonpulmonary infections requiring hospitalization in our cohort. The incidence of infections was compared between SpA and NSBP. Associated risk factors were identified using regression analysis.

## 2 METHODS

Data from December 1995 to June 2019 were retrieved from the Hospital Authority Clinical Management System (CMS), a centralized database established in 1995 of electronic medical records from all public hospitals in Hong Kong. Patients with an expert diagnosis of SpA, AS, SpA with psoriasis (PsO), inflammatory bowel disease (IBD) associated SpA, reactive arthritis (ReA), undifferentiated SpA (uSpA), and human leucocyte antigen (HLA) associated uveitis from 11 rheumatology centers (Queen Mary Hospital, Grantham Hospital, Tung Wah Hospital, Caritas Medical Center, Pamela Youde Nethersole Hospital, Tseung Kwan O Hospital, Queen Elizabeth Hospital, Kwong Wah Hospital, Alice Ho Miu Ling Nethersole Hospital, Pok Oi Hospital, and Prince of Wales Hospital) were included. All patients with NSBP from an orthopedic unit (Queen Mary Hospital) were also included as controls. NSBP was defined as back pain without a specific and identified pathology such as trauma, infection, tumor, deformity, or nerve compression. Patients with coexisting rheumatologic disease in the control group were excluded from analyses. Data were manually retrieved by the author (HYC) and randomly rechecked by another author (SCWC).

The five most prevalent nonpulmonary infections in this cohort were urinary tract infection (UTI), skin infection, gastroenteritis (GE), septic arthritis, and pancreato‐hepatobiliary tract infection. Urinary tract infections included both pyelonephritis and lower urinary tract infection. Skin infection encompassed cellulitis and skin abscess. Pancreato‐hepatobiliary tract infection included liver abscess, pancreatitis, cholangitis, and cholecystitis. Diagnosis was made clinically by the managing physician, with or without confirmatory microbiology results. Date of infection and cultured micro‐organisms were recorded. Mortalities as a result of the mentioned infections were also recorded.

Demographic data included age, sex, ethnicity, and smoking and drinking status. Medications used within one month of first onset of infection were recorded. These include nonsteroidal anti‐inflammatory drug (NSAID), sulfasalazine, methotrexate (MTX), leflunomide, etanercept, infliximab, adalimumab, golimumab, certolizumab, secukinumab, and steroids for greater than 6 months. Comorbidities include history of PsO, IBD, diabetes mellitus (DM), renal impairment, depression, organ transplant, cerebrovascular accident (CVA), human immunodeficiency virus (HIV) infection, intravenous drug use (IVDU), and cirrhosis. Renal impairment was defined as chronic kidney disease Stage 3 or above, according to the Kidney Disease Improving Global Outcomes (KDIGO) 2012 clinical practice guidelines.^12^ CVA included both ischemic and hemorrhagic stroke and excluded transient ischaemic attack (TIA). Antero‐posterior radiographs of the lumbosacral spine were read by one author (SCWC), and sacroiliitis was scored according to the modified New York (mNY) criteria.^13^ Bilateral Grade 2 or unilateral Grade 3 or above was defined as radiographic AS.^13^

The clinical outcomes assessed were the first onset of the five types of nonpulmonary infections. Duration of follow‐up was defined as the time between the initial assessment at the rheumatology or orthopedic clinic and one of the following end‐points: end of the study, day of discharge from the clinic, hospitalization due to infection, and death.

### 2.1 Statistical analyses

Independent *t* test and *χ* ^2^ test were used to compare the continuous and categorical variables respectively between patients with SpA and NSBP. Normality test was done using histograms. Crude incidence rates were reported as the number of the first episode of the respective non‐pulmonary infection per 1000 patient‐years.

Propensity score adjustment method^14^ was used to compare the risk of the five types of nonpulmonary infection between patients with SpA and NSBP. Demographic variables, known or potential risk factors for infection were included in the logistic regression to calculate the propensity score. Factors included in the propensity score calculation were age,^15^ ethnicity,^16^ sex,^16^ smoker,^17^ drinker,^16^ NSAID, steroid for more than 6 months,^18^ DM,^19^ renal impairment,^20^ malignancy,^21^ depression,^22^ history of organ transplant,^23^ CVA,^24^ HIV infection,^25^ IVDA,^26^ and cirrhosis.^27^ Cox regression model was used to determine the risk of individual non‐pulmonary infections in SpA leading to hospitalization after propensity score adjustment.

The mentioned confounding factors, medications, and radiographic AS were also used for screening of potential risk factors of each nonpulmonary infection associated with SpA. This was done by the univariate Cox Regression model. Significant factors with a *p* value of <.1 in the univariate analyses were included in multivariate Cox Regression model using “enter” mode. Apart from NSAID and steroid for more than 6 months, medications included in the univariate Cox regression models were sulfasalazine, methotrexate (MTX), leflunomide, etanercept, infliximab, adalimumab, golimumab, certolizumab, and secukinumab. Results were reported as hazard ratio (HR) with 95% confident interval (CI). All statistics were performed using the International Business Machines Corporation Statistical Package for the Social Sciences (IBM SPSS) package 25.0. Unless specified, a *p* value of less than .05 was defined as statistically significant. Listwise deletion was performed for missing data.

### 2.2 Ethics approval

The study was approved by the ethics committee of the Institutional Review Board of The University of Hong Kong/Hospital Authority Hong Kong West Cluster (reference number UW 18‐263). The Institutional Review Board waived the need for informed consent since personal information had been deidentified in our study. The study was conducted in accordance with the Declaration of Helsinki and the guidance of Good Clinical Practice, November 30, 2006.

## 3 RESULTS

Baseline characteristics of 3018 patients with SpA and 2527 patients with NSBP are shown in Table 1. The SpA group was characterized by younger age, male predominance, history of smoking and drinking, more NSAIDs and steroid use, less comorbidities including DM, renal impairment, and depression, and greater prevalence of skin infection and septic arthritis. The NSBP group had a longer duration of follow‐up. Within the SpA group, most met the criteria for radiographic AS, while IBD‐related SpA was rare. The most common conventional (c‐) and biologic (b‐) DMARDs used were sulfasalazine and etanercept respectively.

**Table 1:** Comparison of baseline characteristics between patients with SpA and NSBP

	Patients with SpA (*n* = 3018)	Patients with NSBP (*n* = 2527)	*p* Value
Demographics			
Age (years) (mean ± SD)	49.6 ± 14.5	62.1 ± 15.0	<.001
Chinese ethnicity	2985/3018 (98.9%)	2487/2527 (98.4%)	.11
Male sex	2058/3018 (68.2%)	914/2527 (36.2%)	<.001
Smoker	886/2975 (29.8%)	455/2422 (18.8%)	<.001
Drinker	245/2975 (8.2%)	155/2422 (6.4%)	.01
Follow‐up duration (years)	9.5 ± 6.0	13.9 ± 5.8	<.001
Medications			
On NSAID	2863/3018 (94.9%)	2120/2527 (83.9%)	<.001
On cDMARDs	1645/3018 (54.5%)	0/2527 (0.0%)	
On SASP	1293/3018 (42.8%)	0/2527 (0.0%)	
On MTX	790/3018 (26.2%)	0/2527 (0.0%)	
On leflunomide	164/3018 (5.4%)	0/2527 (0.0%)	
On bDMARDs	734/3018 (24.3%)	0/2526 (0.0%)	
On etanercept	280/3018 (9.3%)	0/2527 (0.0%)	
On infliximab	107/3018 (3.5%)	0/2527 (0.0%)	
On adalimumab	243/3018 (8.1%)	0/2527 (0.0%)	
On golimumab	201/3018 (6.7%)	0/2527 (0.0%)	
On certolizumab	43/3018 (1.4%)	0/2527 (0.0%)	
On secukinumab	75/3018 (2.5%)	0/2527 (0.0%)	
On steroid >1/2 year	164/3018 (5.4%)	13/2527 (0.5%)	<.001
Comorbidities			
History of psoriasis	655/3018 (21.7%)	0/2527 (0.0%)	<.001
Inflammatory bowel disease	47/3018 (1.6%)	0/2527 (0.0%)	<.001
Radiological AS	1971/2917 (67.6%)	0/2527 (0.0%)	<.001
DM	273/3018 (9.0%)	394/2527 (15.6%)	<.001
Renal impairment (stage 3 or above chronic kidney disease)	187/3018 (6.2%)	329/2527 (13.0%)	<.001
Depression	138/3018 (4.6%)	257/2527 (10.2%)	<.001
History of organ transplant	2/3018 (0.1%)	7/2527 (0.3%)	.052
History of cerebrovascular accident	103/3018 (3.4%)	89/2527 (3.5%)	.83
HIV infection	2/3018 (0.1%)	1/2527 (0.0%)	.67
IV drug addict	1/3018 (0.0%)	4/2527 (0.2%)	.12
Cirrhosis	9/3018 (0.3%)	12/2527 (0.5%)	.29
Infections			
Urinary tract infection	96/3018 (3.2%)	101/2527 (4.0%)	.10
Skin infection	147/3018 (4.9%)	91/2527 (3.6%)	.02
Gastroenteritis	76/3018 (2.5%)	50/2527 (2.0%)	.18
Pancreato‐hepatobiliary tract infection	35/2018 (1.2%)	41/2527 (1.6%)	.14
Septic arthritis	12/3018 (0.4%)	3/2527 (0.1%)	.05

Abbreviations: AS, ankylosing spondylitis; bDMARDs, biological disease‐modifying antirheumatic drugs; cDMARDs, conventional disease‐modifying anti‐rheumatic drugs; DM, diabetes mellitus; HIV, human immunodeficiency virus; IV, intravenous; MTX, methotrexate; NSAID, nonsteroidal anti‐inflammatory drug; NSBP, nonspecific back pain; SASP, sulfasalazine; SD, standard deviation; SpA, spondyloarthritis.

### 3.1 Crude incidence rates and risk of nonpulmonary infections

Crude incidence rates of each of the hospitalized nonpulmonary infections presented in Table 2 show that skin infection (5.3 per 1000 patient‐years) and septic arthritis (0.4 per 1000 patient‐years) were the highest and lowest respectively.

**Table 2:** Crude incidence rates of the hospitalized nonpulmonary infections

	Patients with SpA	Patients with NSBP
Number of UTI	96	101
Patient‐years	28,311.2	34,474.6
Incidence per 1000 patient‐years	3.4	2.9
Number of skin infection	147	91
Patient‐years	27,871.7	34,499.3
Incidence per 1000 patient‐years	5.3	2.6
Number of GE	76	50
Patient‐years	28,296.0	34,746.4
Incidence per 1000 patient‐years	2.7	1.4
Number of pancreato‐hepatobiliary infection	35	41
Patient‐years	28,647.1	34,739.1
Incidence per 1000 patient‐years	1.2	1.2
Number of septic arthritis	12	3
Patient‐years	28,738.9	35,019.0
Incidence per 1000 patient‐years	0.4	0.1

Abbreviations: GE, gastroenteritis; NSBP, nonspecific back pain; SpA, spondyloarthritis; UTI , urinary tract infection.

Propensity score adjusted risks of hospitalized non‐pulmonary infections in SpA with reference to NSBP were: UTI (HR 1.91, 95% CI 1.37–2.65, *p* < .001), skin infection (HR 1.79, 95% CI 1.31–2.43, *p* < .001), GE (HR 1.42, 95% CI 0.94–2.16, p = 1.00), pancreato‐hepatobiliary infection (HR 1.67, 95% CI 0.98–2.83, *p* = .06), and septic arthritis (HR 4.57, 95% CI 1.10–18.98, *p* = .04).

The only nonpulmonary infection related death occurred in the SpA group due to pancreato‐hepatobiliary tract infection. No deaths occurred in the NSBP group.

### 3.2 Risk factors for nonpulmonary infections requiring hospitalization

Cox regression models were built for UTI, skin infection, and septic arthritis—the non‐pulmonary infections found to be more susceptible in patients with SpA. Univariate Cox regression models with UTI as the dependent variable found statistically significant (*p* < .1) associations with age, Chinese ethnicity, male sex, PsO, radiographic AS, infliximab, steroid therapy greater than 6 months, chronic renal impairment, malignancy, and CVA. Multivariate Cox regression analysis found Chinese ethnicity, male sex, infliximab, chronic renal impairment, and CVA were independent risk factors (Table 3).

**Table 3:** Cox regression analyses using urinary tract infection as dependent variable

	Urinary tract infection			
	Univariate Cox regression models		Multivariate Cox regression	
Characteristics	Hazard ratio (95% CI)	*p* Value	Hazard ratio (95% CI)	*p* Value
Age	1.03 (1.02–1.05)	<.001	1.01 (1.00–1.03)	.16
Chinese ethnicity	0.22 (0.07–0.71)	.01	0.24 (0.07–1.80)	.02
Male sex	0.57 (0.38–0.85)	.01	0.64 (0.42–0.99)	.04
Smoker	0.71 (0.44–1.13)	.15		
Drinker	1.29 (0.67–2.49)	.44		
History of psoriasis	1.64 (1.08–2.51)	.02	1.06 (0.62–1.79)	.84
History of inflammatory bowel disease	1.42 (0.35–5.77)	.62		
Radiographic axial SpA	0.61 (0.40–0.93)	.02	0.71 (0.43–1.16)	.17
NSAID	0.64 (0.28–1.46)	.29		
Sulfasalazine	0.71 (0.47–1.07)	.10		
Methotrexate	1.33 (0.88–2.02)	.18		
Leflunomide	0.81 (0.33–1.99)	.65		
Etanercept	1.15 (0.60–2.21)	.68		
Infliximab	1.88 (0.93–3.82)	.08	2.21 (1.05–4.67)	.04
Adalimumab	0.43 (0.14–1.35)	.15		
Golimumab	1.12 (0.52–2.42)	.77		
Certolizumab	0.76 (0.11–5.45)	.78		
Secukinumab	1.21 (0.38–3.83)	.74		
Steroid for > ½ year	1.91 (1.02–3.58)	.04	1.48 (0.76–2.91)	.25
Diabetes mellitus	1.54 (0.89–2.68)	.12		
Chronic renal impairment	4.04 (2.54–6.42)	<.001	2.61 (1.50–4.52)	.001
Malignancy	1.98 (1.00–3.94)	.05	0.89 (0.41–1.93)	.77
Depression	1.71 (0.83–3.52)	.15		
History of organ transplant	0.05 (0.00 to >999)	.86		
Cerebrovascular accident	4.00 (2.30–6.94)	<.001	2.63 (1.37–5.05)	<.01
HIV infection	0.50 (0.00 to >999)	.90		
IV drug addict	0.50 (0.00 to >999)	.90		
Cirrhosis	0.50 (0.00 to >999)	.69		

Abbreviations: CI, confidence interval; HIV, human immunodeficiency virus; IV, intravenous; NSAID,  nonsteroidal anti‐inflammatory drug; SpA, spondyloarthritis.

In the univariate Cox regression models using skin infection as dependent variables, smoker, history of PsO, sulfasalazine, methotrexate, leflunomide, adalimumab, steroid for more than 6 months, DM, chronic renal impairment, history of organ transplant, and CVA had significant associations (*p* < .1). Multiple Cox regression analysis showed smoking, history of PsO, steroids for more than 6 months, and chronic renal impairment were independent risk factors for skin infection leading to hospitalization in SpA (Table 4).

**Table 4:** Cox regression analyses using skin infection as a dependent variable

	Skin infection			
	Univariate Cox regression models		Multivariate Cox regression	
Characteristics	Hazard ratio (95% CI)	*p* Value	Hazard ratio (95% CI)	*p* Value
Age	1.00 (0.99–1.01)	.81		
Chinese ethnicity	1.12 (0.16–8.03)	.91		
Male sex	1.22 (0.85–1.75)	.28		
Smoker	1.66 (1.19–2.31)	<.01	1.81 (1.30–2.53)	<.001
Drinker	1.28 (0.75–2.18)	.37		
History of psoriasis	2.87 (2.07–3.97)	<.001	2.47 (1.66–3.67)	<.001
History of inflammatory bowel disease	0.44 (0.06–3.13)	.41		
Radiographic axial SpA	1.14 (0.78–1.66)	.49		
NSAID	0.75 (0.37–1.53)	.43		
Sulfasalazine	0.75 (0.54–1.04)	.08	0.84 (0.59–1.21)	.35
Methotrexate	1.62 (1.17–2.26)	<.01	1.06 (0.71–1.59)	.78
Leflunomide	2.05 (1.23–3.39)	.01	1.08 (0.61–1.93)	.78
Etanercept	1.36 (0.83–2.23)	.22		
Infliximab	1.58 (0.78–3.20)	.20		
Adalimumab	2.34 (1.45–3.79)	.001	1.62 (0.97–2.70)	.07
Golimumab	0.92 (0.47–1.81)	.81		
Certolizumab	0.98 (0.24–3.95)	.98		
Secukinumab	1.39 (0.57–3.39)	.47		
Steroid for > ½ year	2.58 (1.63–4.11)	<.001	2.22 (1.38–3.59)	<.01
Diabetes mellitus	1.72 (1.11–2.66)	.02	1.11 (0.70–1.75)	.67
Chronic renal impairment	2.52 (1.63–3.87)	<.001	1.82 (1.14–2.90)	.01
Malignancy	1.27 (0.65–2.49)	.49		
Depression	1.13 (0.55–2.30)	.75		
History of organ transplant	9.04 (1.26–64.76)	.03	7.05 (0.95–52.38)	.06
Cerebrovascular accident	1.79 (0.96–3.31)	.07	1.21 (0.63–2.31)	.57
HIV infection	0.50 (0.00 to >999)	.87		
IV drug addict	0.50 (0.00 to >999)	.87		
Cirrhosis	1.91 (0.27–13.65)	.52		

Abbreviations: CI, confidence interval; HIV, human immunodeficiency virus; IV, intravenous; NSAID,  nonsteroidal anti‐inflammatory drug; SpA, spondyloarthritis.

In the univariate Cox regression models using septic arthritis as dependent variables, sulfasalazine, adalimumab, and malignancy had significant associations (*p* < .1). Multivariate Cox regression analysis showed only malignancy was the independent risk factor for septic arthritis leading to hospitalization in SpA (Table 5).

**Table 5:** Cox regression analyses using septic arthritis as a dependent variable

	Septic arthritis			
	Univariate Cox regression models		Multivariate Cox regression	
Characteristics	Hazard ratio (95% CI)	*p* Value	Hazard ratio (95% CI)	*p* Value
Age	1.01 (0.96–1.05)	.81		
Chinese ethnicity	20.32 (0.00 to >999)	.85		
Male sex	2.35 (0.52–10.73)	.27		
Smoker	1.15 (0.35–3.82)	.82		
Drinker	1.02 (0.13–7.88)	.99		
History of psoriasis	0.30 (0.04–2.34)	.25		
History of inflammatory bowel disease	0.05 (0.00 to >999)	.77		
Radiographic axial SpA	1.96 (0.43–8.98)	.38		
NSAID	21.44 (0.00 to >999)	.64		
Sulfasalazine	3.16 (0.85–11.70)	.09	2.91 (0.77–10.96)	.11
Methotrexate	1.16 (0.35–3.87)	.81		
Leflunomide	1.37 (0.18–10.58)	.77		
Etanercept	0.04 (0.00–216.35)	.47		
Infliximab	1.68 (0.22–13.05)	.62		
Adalimumab	3.89 (1.05–14.38)	.04	3.61 (0.95–13.71)	.06
Golimumab	1.35 (0.17–10.46)	.77		
Certolizumab	0.05 (0.00 to >999)	.79		
Secukinumab	3.35 (0.43–26.04)	.25		
Steroid for > ½ year	1.29 (0.17–9.98)	.81		
Diabetes mellitus	1.70 (0.37–7.75)	.50		
Chronic renal impairment	0.04 (0.00–459.19)	.51		
Malignancy	3.75 (0.82–17.16)	.09	4.74 (1.02–22.08)	.05
Depression	1.74 (0.22–13.47)	.60		
History of organ transplant	0.50 (0.00 to >999)	.95		
Cerebrovascular accident	0.05 (0.00 to >999)	.62		
HIV infection	0.05 (0.00 to >999)	.96		
IV drug addict	0.05 (0.00 to >999)	.97		
Cirrhosis	0.50 (0.00 to >999)	.89		

Abbreviations: CI, confidence interval; HIV, human immunodeficiency virus; IV, intravenous; NSAID,  nonsteroidal anti‐inflammatory drug; SpA, spondyloarthritis.

### 3.3 Microorganisms identified

The three most prevalent micro‐organisms found in each type of infection were similar in both groups, except for pancreato‐hepatobiliary tract infection in which *Escherichia coli* was more prevalent in NSBP (Table 6).

**Table 6:** Microorganisms identified in different types of hospitalized nonpulmonary infections

	Patients with SpA	Patients with NSBP	*p* Value
Urinary tract infection (urine culture)			
*Escherichia coli*	43/96 (44.8%)	49/101 (48.5%)	.60
*Enterococcus*	5/96 (5.2%)	6/101 (5.9%)	.82
*Proteus*	3/96 (3.1%)	6/101 (5.9%)	.34
Skin infection (wound swab for culture)			
*Staphylococcus aureus*	33/147 (22.4%)	19/91 (20.9%)	.78
*Pseudomonas aeruginosa*	5/147 (3.4%)	2/91 (2.2%)	.59
*Streptococcus pneumoniae*	4/147 (2.7%)	3/91 (3.3%)	.80
Gastroenteritis (stool culture)			
*Vibrio cholerae*	2/76 (2.6%)	4/50 (8.0%)	.17
*Campylobacter jejuni*	2/76 (2.6%)	1/50 (2.0%)	.82
*Norovirus*	2/76 (2.6%)	0/50 (0.0%)	.25
Hepatobiliary tract infection (bile culture)			
*Klebsiella pneumoniae*	8/35 (22.9%)	4/41 (9.8%)	.12
*Escherichia coli*	1/35 (2.9%)	8/41 (19.5%)	.03
*Enterococcus faecalis*	2/35 (5.7%)	2/41 (4.9%)	.87
Septic arthritis (tissue culture)			
*Staphylococcus aureus*	3/12 (25.0%)	1/3 (33.3%)	.77
*Streptococcus pneumoniae*	2/12 (16.7%)	0/3 (0.0%)	.45

Abbreviations: NSBP, nonspecific back pain; SpA, spondyloarthritis.

### 3.4 Missing values

Missing values constituted less than 5% of the study population, and hence inconsequential. Out of a total of 5545 patients, radiographs of the lumbosacral spine were missing in 102 (1.8%), and smoking and drinking status were missing in 148 (2.7%).

## 4 DISCUSSION

This study of real‐world data in SpA showed increased risk of hospitalized non‐pulmonary infections including UTI, skin infection, and septic arthritis. Rheumatic disease and treatment‐specific independent risk factors for skin infection were PsO and steroids, and infliximab for UTI.

In general, rheumatic disease predisposes one to a higher risk of infection. A population‐based study showed that the incidence of hospitalized infection in patients with rheumatoid arthritis (RA) was 9/100 person‐years, compared to 5/100 in those without RA.^28^ Similarly, the overall risk of infection was also increased in systemic lupus erythematosus.^29^ Without exception, this study in SpA has also found an increased adjusted risk of UTI, skin infection, and septic arthritis.

The pathogenesis of infection in SpA includes immunologic and physical factors. T helper (Th)−17 dysregulation, thought to be the central role in the pathogenesis of SpA,^30^ results in decreased production and recruitment of neutrophils, predisposing individuals to extracellular bacterial infection. Regulatory T cells (Tregs) and CD8‐memory cells are also dysregulated.^30^ In patients with AS, Tregs were found to be significantly higher in synovial fluid than in peripheral blood,^31^ attenuating the proliferation of effector T cells.^32^ Little is known regarding the effects of such alteration in T cell compositions. It is possible that the attenuated effector T cells response may result in impaired host defense against infections. Urinary stasis as a result of immobility was shown to increase the risk of UTI^33^ while joint pathology is associated with septic arthritis.^34^ The microorganisms identified were common causes of infections in their respective sites. There were no significant differences in the pathogens between SpA and NSBP, except in hepatobiliary infections. *E. coli* and *Klebsiella pneumoniae* are both common causes of hepatobiliary infections. HLA‐B27 has been linked to the perpetuation of *Klebsiella* infection and the pathogenesis of ankylosing spondylitis.^35^ This might explain the observation of a higher frequency of *E. coli* in causing hepatobiliary infection among patients with NSBP compared with SpA.

From this data, one fatality from infection occurred in the SpA group, despite a significant proportion on immunosuppressant therapy (54.5% on cDMARDs and 24.3% on bDMARDs). A meta‐analysis of RCTs in AS found low risk of serious infection (4 per 1000 patient‐years) on anti‐TNF drugs compared with placebo.^36^ Real‐life data from a Canadian longitudinal observational cohort of 440 patients with SpA also showed a slight risk of serious infection.^37^ This study had similar findings.

Infliximab was the only DMARD found to be associated with increased risk (2.2 times compared with control) of hospitalized UTI, among other potential variables analyzed including steroids and SpA subtypes. Previous research has also consistently found that among immunosuppressants, the greatest risk of infection occurred with infliximab.^38^, ^39^ Pharmacodynamic properties unique to infliximab^40^ that results in downregulation of interferon (IFN)‐γ was not observed with etanercept.^40^ IFN‐γ has an important role in both innate and adaptive immunity by enhancing natural killer cells, activating macrophages, and inducing expression of major histocompatibility complex (MHC) Class II molecules. Pharmacokinetically, high peak serum concentrations of infliximab up to 50 times the median trough level^41^ also predisposes to infection.^42^ Pneumonia,^11^ tuberculosis,^10^ and cutaneous herpes zoster^9^ were also increased with infliximab therapy. Infliximab may have distinct immunosuppressive effects that increase infection risk.

Steroid therapy for more than six months was associated with an increased risk (2.2 times compared with control) of skin infection. The immunosuppressive properties of steroids involve multiple pathways. Suppression of activation and differentiation of macrophages decreases downstream production of IL‐1, IL‐6, TNF, prostaglandins, and leukotrienes.^43^ Steroid‐induced lymphopenia occurs via inhibition of T‐cell activation by inhibition of IL‐2, IL3, IL‐4, and IL‐6,^43^ and suppression of dendritic cell maturation and function.^44^ Long‐term steroid therapy has been shown to increase the risk of community‐acquired pneumonia^11^ and tuberculosis^10^ in SpA. Prolonged steroid therapy in SpA is not recommended^45^, ^46^, ^47^ by international consensus guidelines.

PsO was an independent risk factor for skin infection, with 2.5 times increased risk compared to control. Akin to SpA, patients with PsO are more susceptible to infection as a result of immune dysregulation and disruption of skin barrier function. A population‐based study in the United States showed that PsO is associated with an increased risk of a range of infections including cellulitis, infectious arthritis, viral infection, tuberculosis, osteomyelitis, meningitis, encephalitis, septicemia, and enterocolitis.^48^ In addition, hospitalization rates in PsO^49^ were affected by socioeconomic factors, suggesting inadequate treatment could result in an increased risk of infection.

In our study, the increased risk of septic arthritis in SpA was not due to DMARD therapy. Other conditions involving the joints such as osteoarthritis (OA) and RA also had an increased risk of infectious arthritis.^34^ Although the prevalence in our cohort was lower than that of RA,^50^ septic arthritis in SpA is likely to have similar pathogenesis. The increased risk of septic arthritis with malignancies may be indirectly related to treatment such as chemotherapy and immunotherapy, which was not addressed in this study. Given the small number of cases with septic arthritis, the current study did not have adequate power to detect the effect of age, DM, and alcohol.^34^ Other potential risk factors including joint deformity, disease activity, and functional impairment were not recorded in the electronic database.

Other risk factors for non‐pulmonary infections identified in the present study were consistent with previous findings. Non‐Chinese ethnicity,^16^ female gender,^16^ chronic renal impairment,^20^ and CVA^24^ were risk factors for UTI; smoking,^17^ and chronic renal impairment^20^ for skin infection; and malignancy^21^ for septic arthritis. Although diagnostic inaccuracies may occur, microorganisms identified were similar in both SpA and NSBP groups. This suggests that infection in SpA likely shares similar modes of transmission as the general population.

### 4.1 Limitations and future perspectives

Nonspecific back pain was selected as a surrogate for the general population, which may be less than ideal. Patients with NSBP were selected for their immunocompetency, and absence of obvious additional risk factors for nonpulmonary infections, in which comprehensive medical records were available. Other limitations stem from its retrospective nature. The diagnosis of infection was based on clinical findings, with or without confirmatory microbiology tests. However, similar percentages with missing microbiological results were found in both groups, and hence similar margin of error. To minimize bias, propensity score adjustments were done to enhance validity of comparisons, however, these did not include disease activity, joint damage, functional status, and body weight. Therefore, the study could not account for all potential confounding factors, and could only provide insights into associations between conditions. Similarly, our study did not include PsO disease severity and therefore could not conclude whether adequately treated patients would be at lower risk of infections. Recurrence of infections and mortality were not included, which would otherwise provide a broader context of risk over time. As the data encompassed over 24 years, sensitivity analysis was used to adjust for temporal advances in treatment. As mentioned previously, some of the clinical outcomes (e.g. septic arthritis) were small. There were also few patients on the newer bDMARDs (e.g., secukinumab), therefore the effect of these on the risk of infections is unknown. A large prospective cohort to assess disease activity, chronicity, and functional impairment of SpA could provide answers to the unresolved questions.

## 5 CONCLUSION

SpA was associated with an increased risk of UTI, skin infection, and septic arthritis compared with NSBP. Independent risk factors include infliximab, duration of steroid therapy greater than six months, and PsO. Rheumatologists should consider aim to avoid long‐term steroid to help prevent nonpulmonary infections in SpA. Further studies are needed to ascertain the relationship between disease severity and the risk of major infections. Monitoring for UTI may be appropriate with the use of infliximab.

## CONFLICTS OF INTEREST

The authors declare no conflicts of interest.

## Data Availability

Data are available from Dr Ho Yin Chung upon reasonable request.
